# Notes and News

**Published:** 1902-03-29

**Authors:** 


### NOTES AND NEWS.

The King recently received the members of the Advisory Committee who are to select the site for the sanatorium for tuberculosis. The committee are: Sir William Broadbent, Sir R. Douglas Powell, Sir Francis Laking, Sir Felix Semon, Dr. Theodore Williams, and Dr. Gustav Besolde. The competitive plans and essays by medical men are, we believe, now handed in. It is to be hoped that the interested public may have the opportunity of seeing plans with special features which may be the outcome of the competition, though they may not be selected.

The King has appointed a Royal Commission to inquire into the opportunities in State-aided day schools for physical training. The proposed training will be considered in connection with continuation classes.

The Prince and Princess of Wales visited the Hospital for Sick Children, Great Ormond Street, last week.

The Prince of Wales will open the New Alexandra Hospital at Rhyl on the occasion of his visit to Carnarvon to be installed as Chancellor of the Welsh University.

Asiatic cholera has broken out in Manila. The death rate is a very large one. It is believed that the disease has been introduced from Hong Kong.

A fête has just been held at Cannes in aid of the English Hospital. The British colony was largely represented, and the hospital will receive substantial benefit.

What is the matter with Portsmouth? A leaflet is being circulated which by imputation accuses the hospital authorities of many enormities, but so irresponsible is the person or persons from whom the leaflet emanates that it bears no inscription by which its accusations can be identified with any particular hospital in the United Kingdom. The circular is headed "A Hospital Scandal," but the want of business methods at Portsmouth is evidently not confined to the hospital at any rate. Is it in the Portsmouth air?

The mortality from plague in the Punjaub is very great at the present time, larger than has been known. The policy of non-interference and non-compulsion amongst the natives is held to be responsible for the present state of things.

The decision in the case of the gifts of the late Mrs. Dowling to her medical attendant must cause the stability of any gifts of money received from persons during illness to be a matter of question. The persons who accept money given from motives of gratitude by a sick person will be wiser to wait and see whether the gift is likely to be contested before they spend it. It will not be so easy in the future to retain gifts unless the recipient can prove that the giver had proper advice as to making the gift, for this absence of advice has deprived the doctor in question of the large sums of money Mrs. Dowling had given him, and which he must return to the executors.

In connection with the present outbreak of small-pox in the Metropolis, the numbers of which have been otherwise than small in comparison to other visitations, the enormous expense to which the rate-payers are being put to provide hospital accommodation must create some consternation. £400,000 will be required to defray the expenditure on temporary buildings and ambulance stations alone. Mr. Helby, the Chairman of the Finance Committee, does not at all hold out a hope that this sum will be a final payment required on behalf of this epidemic. At the meeting at which these statements were made Sir E. Galsworthy maintained that every penny spent on small-pox provision was justifiable.

Last week we announced the large amount of support which is being given to cancer investigation in Germany. We are now glad to be able to report that substantial encouragement is being given in the same direction in England. The Cancer Research Fund has received three munificent donations of £5,000 each, one of which is anonymous, one comes from Mr. H. L. Bischoffsheim, who for years has maintained the Street Ambulance Organisation of London, and the third is given by the Goldsmiths' Company as a special memorial of the Coronation. This donation is accompanied by a stipulation that it shall be under the control of the Colleges of Physicians and Surgeons.

The annual financial report of the Metropolitan Asylums Board should be an interesting object-lesson to those who are under the impression that State control for hospitals would secure uniformity of efficiency and cost. The Metropolitan Asylums Board does its work very admirably, but not inexpensively, and a perusal of the financial statement shows, as Mr. Helby, the chairman of the Finance Committee, points out, that there is a considerable discrepancy of expenditure at the various hospitals under the Board. Mr. Helby holds that the sub-committees are responsible for such economy and also for such wastefulness as prevails, and thinks there should be more central observation of the working of the institutions. This would certainly stimulate both economy and efficiency.

